# Lymphocyte-Predominant Hodgkin's Disease in Children: A Case Study and Review of the Literature

**DOI:** 10.1155/2015/351431

**Published:** 2015-03-24

**Authors:** James R. Stier, Robert J. Vasquez

**Affiliations:** ^1^Department of Pediatrics, Tulane University School of Medicine, New Orleans, LA 70112, USA; ^2^Department of Pediatrics, Ochsner Health System, New Orleans, LA 70112, USA; ^3^Department of Pediatrics, Tulane University School of Medicine, New Orleans, LA 70112, USA; ^4^Department of Pediatric Hematology-Oncology, Ochsner Health System, New Orleans, LA 70121, USA; ^5^University of Queensland School of Medicine, Brisbane, QLD 4006, Australia

## Abstract

A three-year-old boy presented with an enlarging neck mass. Biopsy demonstrated IgD-positive nodular lymphocyte-predominant Hodgkin lymphoma (NLPHL), which was staged as IIa. The patient received cyclophosphamide, doxorubicin, vincristine, and prednisone (CHOP) with rituximab and had excellent results. NLPHL is a relatively rare disease that is biologically distinct from classic Hodgkin lymphoma (cHL). NLPHL is a B-cell malignancy likely of germinal center origin that has an overall good prognosis and favorable response to treatment. Unlike cHL, NLPHL is ubiquitously CD20-positive. Recent evidence supports the efficacy of targeted anti-CD20 therapy in NLPHL, though prospective data is limited. This case demonstrates several unique features of NLPHL and further supports the use of rituximab in front-line therapy. The clinical characteristics among patients at various ages are discussed with a special focus on the IgD-positive subtype. A thorough literature search demonstrates this to be the youngest patient with NLPHL yet described.

## 1. Case

A three-year-old boy originally from Saudi Arabia was referred to ENT clinic with a five-week history of an enlarging right neck mass associated with fevers, night sweats, and fatigue. Physical exam revealed a roughly 2.5 × 4 cm slightly tender, mobile, matted lymph node located inferior to the sternocleidomastoid with several smaller cervical lymph nodes palpated bilaterally. The patient was given a course of oral antibiotics and though his systemic symptoms quickly resolved the neck mass persisted. He subsequently underwent excisional biopsy of the mass. Surgical dissection revealed a 6 × 3 × 3 cm node adherent to the internal jugular vein. The node and several surrounding satellite lymph nodes were removed and sent for pathologic analysis.

Flow cytometry was normal with 96% “unremarkable” T-cells and 1.8% blasts. EBV and AFB stains were negative. The specimens were sent to two major pathology centers for further study. Final interpretation from both consulting institutions was consistent with* nodular lymphocyte-predominant Hodgkin lymphoma* (NLPHL) with morphology demonstrating atypical lymphoid cells staining positive for CD20, IgD, CD45, OCT-2, PAX-5, and bcl-6 but negative for CD15 and CD30. Architecturally, the tissue featured nodular areas consisting of a CD21-positive follicular dendritic cell meshwork with areas of more diffuse growth amidst a T-cell-rich background—all features resembling those previously described in typical IgD-positive NLPHL.

**Figure 1:**
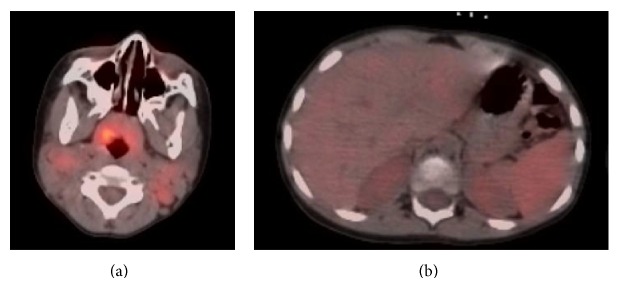
PET/CT demonstrates increased tracer uptake within bilateral cervical lymph nodes and the nasopharyngeal region (often normal in children). Slightly increased uptake in the spleen is seen in the image on the right with no apparent focal lesion.

PET/CT (Figure 1) demonstrated increased tracer uptake within bilateral cervical lymph nodes (estimated SUV of 4.5) and spleen (estimated SUV of 3.6). Bone marrow biopsies showed no morphologic evidence of metastatic disease. Staging was discussed with several international Hodgkin experts who agreed that splenic disease without involvement of thoracic or intra-abdominal lymph nodes would be unlikely in NLPHL particularly without focal areas of hypermetabolic activity within the spleen; however, we could not completely discount this finding and it factored into our therapy plan. B-symptoms are unusual in NLPHL and since the patient's systemic symptoms resolved with antibiotics and did not recur in the intervening weeks prior to initiation of chemotherapy, we concluded that the constitutional symptoms were likely due to an unrelated infectious process. The lymphoma was therefore staged as IIa.

The patient underwent three cycles of chemotherapy with cyclophosphamide, doxorubicin, vincristine, and prednisone with rituximab (R-CHOP) at standard dosing (cyclophosphamide 800 mg/m^2^, doxorubicin 50 mg/m^2^, and vincristine 1.4 mg/m^2^ on days 1 and 8, prednisone 40 mg/m^2^/day for 7 days, and rituximab 375 mg/m^2^). Rituximab was added due to presence of CD20 positivity and as a way of amplifying therapy, given the uncertainty of splenic involvement, while hopefully not increasing risk for late effects. The patient tolerated chemotherapy well with no admissions for fever and neutropenia and no significant toxicities. Repeat PET/CT scans performed at one month and five months following completion of chemotherapy showed complete resolution of all hypermetabolic activity. Eight months after completion of therapy, the family moved back to Saudi Arabia, and a pediatric oncologist in that region subsequently assumed the patient's care. As of this writing, the patient is doing well with no evidence of recurrence at more than 2 years after therapy.

## 2. Overview

Nodular lymphocyte-predominant Hodgkin lymphoma (NLPHL) accounts for approximately 5% of all cases of Hodgkin lymphoma [1]. Histologic and molecular features of NLPHL demonstrate it to be a unique entity from classic Hodgkin lymphoma (cHL). NLPHL typically has an indolent clinical course often characterized by multiple late relapses. Despite this propensity for relapse, the prognosis of NLPHL following treatment compares favorably to cHL and survival following relapse is also quite high [2]. However, NLPHL has a notable predilection toward developing into diffuse, large B-cell non-Hodgkin's lymphoma (DLBCL) in as many as 12% of cases [3].

## 3. Presentation 

Patients with NLPHL typically present with early-stage disease. In the German Hodgkin Study Group (GHSG), 63% of patients with NLPHL were diagnosed in early stages compared with 22% of cHL patients [4]. Among pediatric patients, NLPHL is more common among preadolescent patients and occurs in an approximate male to female ratio of 5 to 1 [5]. The disease constitutes roughly 10–20% of Hodgkin lymphoma in children 14 years of age or younger compared with 5–8% in patients aged 15–19 [6]. A case series of 55 children with NLPHL found the median age of presentation among pediatric patients to be 13 years (ranging from 4 to 17 years in this particular study) [7].

Nodal involvement tends to be peripheral, most often involving the cervical, inguinal, and axillary regions. Mediastinal involvement is uncommon with an incidence of 8% [8]. Organ involvement is relatively rare as well with 8% of patients having spleen, 6% liver, 2% lung, and 1% skeletal involvement [2].

There is often a long lag between initial detection of an enlarged lymph node and diagnosis of NLPHL [9]. The possible delay in detection may be due to the notable lack of B-symptoms in most patients with NLPHL. Only 9% of NLPHL patients in the GHSG presented with a history of B-symptoms, compared with 40% of patients with cHL [4].

## 4. Diagnosis 

Pathologically, NLPHL is characterized by a nodular proliferation of large neoplastic cells that express CD20. The hallmark of NLPHL is the LP (“lymphocyte-predominant”) cell. Previously known as the L&H (“lymphocytic and histiocytic”) cell due to the belief that it was of histiocytic origin, the LP cell is now known to be of B-cell lineage [10]. The cell is also occasionally referred to as the “popcorn cell” due to its resemblance to a kernel of popcorn. Malignant LP cells in NLPHL constitute around 1% of the total lymph node mass [11]. The nodular appearance likely represents transformation of germinal centers, and at least a partially nodular pattern is required for diagnosis according to the current WHO definition [12].

IgD expression among LP cells in NLPHL (as noted in our patient) has been rarely described in the literature. One large case series looked at 180 patients with NLPHL, of whom 48 (27%) demonstrated IgD positivity [13]. The IgD-positive cases were more likely to demonstrate LP cells in an extrafollicular distribution (69%) than were IgD-negative cases (13%). The IgD-positive cases also presented at a younger median age (21 versus 44 years) and had an even stronger male predominance (23 to 1 versus 1.5 to 1). None of the IgD-positive cases showed coexpression of IgM or IgG but 93% were positive for the transitional B-cell surface protein CD38. The distribution of affected lymph nodes in this study was cervical > inguinal > axillary in IgD-positive cases versus axillary > inguinal > cervical in IgD-negative disease. This study concluded that cases of IgD-positive NLPHL do not differ from IgD-negative cases regarding cellular derivation and most other immunophenotypic characteristics but do exhibit distinctive clinical features and more often involve the interfollicular region in a background relatively rich in T-cells [13].

Another review of 302 pediatric and adult patients also showed that IgD positivity was significantly more common in pediatric patients (70% versus 38% in adults). The male to female ratio was 16 to 1 in IgD-positive cases with a median age of 14 years for IgD-positive patients versus 21 for IgD-negatives [14]. A match-paired analysis from the GHSG database also supported a strong propensity toward male sex and younger age in IgD-positive patients [15].

## 5. Prognosis

The overall prognosis of NLPHL compares favorably to cHL. One long-term observational study followed 164 patients in France between 1973 and 2003 [3]. In this study, the 5-year and 10-year progression-free survival (PFS) rates were 81% and 60%, respectively, for the 106 (65%) patients who underwent treatment. Fifty-eight (35%) patients were followed with a watch-and-wait strategy. Among this group of patients, progression-free survival rates were 59% and 41%, respectively. While progression was more common in the watch-and-wait group, overall survival at 5 and 10 years did not differ significantly between the two groups. Limitations of this study include the long 30-year time period for enrollment, which resulted in a wide range of treatment modalities. Furthermore, the median age of participants was 30 years at the time of enrollment (range: 8 to 64 years) possibly decreasing validity in young and elderly patients.

In a large retrospective analysis of 8,298 HL patients treated within 8 prior GHSG studies (performed between 1988 and 2002), NLPHL had a small but statistically significant advantage in overall survival and freedom from treatment failure (OS 96% versus 92%, *P* = 0.0116; FFTF 88% versus 82%, *P* = 0.0093). The included studies enrolled a total of 394 patients with NLPH and 7,904 with cHL and excluded patients younger than 16 [16]. Despite limited data on pediatric outcomes, survival in NLPHL has been generally shown to be excellent with overall survival at or near 100% in most studies [10].

Few histopathologic factors impacting prognosis have been elucidated. A small matched-paired analysis from the GHSG database did not show significant differences in relapse rate with expression of IgD, CD15, p-STAT6, ICOS, or those positive for Epstein-Barr virus. However, there was a slightly lower relapse rate among patients presenting with a pattern of epithelioid cell clusters [15]. Fan and colleagues investigated the pathologic appearance of 77 cases of NLPHL from patients aged 4 to 91 years and showed that a pattern resembling diffuse T-cell-rich B-cell lymphoma was significantly more common in cases of recurrence [17]. A review of 423 NLPHL cases from 9 GHSG trials showed that histopathologic variation characterized by lymphoma cells outside of the B-cell nodules or overall B-cell depletion was associated with advanced disease (29.5% versus 14.6%, *P* = 0.0012) and a higher relapse rate (18.1% versus 6.5% at 5 years, *P* = 0.0009) [18].

## 6. Treatment 

Treatment for NLPHL is evolving and most evidence for therapy is based on retrospective studies. The view of NLPHL as a subtype of cHL has historically led to treatment strategies that have been similar or identical between these two diseases, though they are now increasingly recognized as distinct.

Almost all patients now receive a relatively short (often 6–9 weeks) course of chemotherapy with or without radiation [11]. Therapy of Stage I disease, however, may involve surgical resection of the tumor only, which in two studies had an overall survival rate of 100% but an event-free survival of 57% and 69% at 43 and 70 months, respectively [19, 20].

The current role of low-dose involved-field radiation therapy (LD-IFRT) is an area of ongoing debate and investigation. So far, the relatively few prospective and controlled randomized trials comparing chemotherapy alone to LD-IFRT with chemotherapy have demonstrated a superior event-free survival among the combined modality treatment groups while overall survival has not differed significantly [21].

In fact, overall NLPHL survival with treatment was 100% in each of five prospective studies published between 2007 and 2012. These studies enrolled between 26 and 81 patients, with three comparing chemotherapy to chemotherapy-plus-radiation treatment groups. Length of followup ranged from 5 to 10 years. Chemotherapy regimens used in these studies were diverse and included COPP/ABV, CVP, VAMP, and DBVE [7, 19, 22–24].

There are currently two large ongoing multicenter studies to help elucidate the preferred treatment modality. A German study by Koerholz and colleagues compares three treatment groups—surgery alone, surgery plus CVP, and CVP alone. A Children's Oncology Group study employs a stepwise therapeutic approach involving tumor resection followed by observation for 6-7 weeks. Patients with evidence of residual disease after observation then receive CHOP every three weeks for a total of three cycles. Patients with residual disease following chemotherapy then proceed to involved-field radiation.

A few studies are also investigating the use of rituximab, an anti-CD20 monoclonal antibody, which has shown promise as a therapeutic option in NLPHL and has become near-standard therapy for relapsed disease. In a prior retrospective analysis of 88 cases of NLPHL over a 30-year period, 8 patients received rituximab during the course of their treatment, though none as first-line therapy. There were no cases of relapse in the 4 patients who received rituximab as second-line therapy, while 2 of 4 patients who received rituximab as part of 3rd- through 5th-line therapy ultimately relapsed. The median followup for these 8 patients was 30 months [25].

A prospective study of 15 patients with relapsed or refractory NLPHL demonstrated response in 94% of patients treated with rituximab monotherapy with 8 patients achieving complete remission and 6 achieving partial remission [26]. A prospective study of 28 newly diagnosed patients showed response in 100% with 24 patients achieving complete remission and 4 achieving partial remission with rituximab monotherapy [1].

One study by Advani and colleagues evaluated the time course for treatment with rituximab. This prospective study of 39 patients with NLPHL compared one course of rituximab monotherapy (375 mg/m^2^) weekly for 4 weeks with the same dose of rituximab for 4-week cycles repeating every 6 months for a total of 2 years. Twenty-three patients (12 new diagnoses and 11 previously relapsed) in the one-month treatment group were compared to 16 patients (9 new diagnoses and 7 previously relapsed) in the cycled-therapy group. Overall, 67% of all patients achieved complete remission and 33% partial remission after the initial 4 weeks of rituximab monotherapy. At the end of the study, estimated 5-year PFS was 39.1% for the one-month treatment group versus 95.7% for the cycled treatment group. The study supported an approximate 3-year increase in length of PFS by the cycled protocol in both first-line and relapsed settings, though the difference was not statistically significant likely due to the small number of enrolled patients [27].

## 7. Conclusion

NLPHL remains a relatively rare disease with a good overall prognosis among both children and adults. In this paper, we report the case of a 3-year-old boy with newly diagnosed NLPHL (to our knowledge, the youngest case ever reported in the literature) who achieved complete remission of his Stage IIa disease with combined cytotoxic chemotherapy and the anti-CD20 antibody rituximab.

The outcome of this case supports the use of rituximab with traditional chemotherapy regimens as first-line, upfront therapy for NLPHL—though prospective studies are needed to confirm the added benefit of this combined-treatment approach. The prognosis and clinical significance of IgD-positive disease are another area that warrants further investigation. Answering these clinical questions will take a concerted effort and cooperation of multiple centers given the relatively low incidence of NLPHL.
